# Indents

**Published:** 1901-07-15

**Authors:** 


### INDENTS.

☞ **Brief Articles of Technical or Scientific Value will receive notice and comment. Contributions invited.**

An Improvised Jack-screw.—In a case where a long Jack-screw was needed, I utilized a disk mandrel. The head was ground square and the shank cut to the proper length, making a very effective Jack-screw.—W. H. Mitchell, in *Dental Cosmos.*

Another Toothache Cure.—A man in Pennsylvania recently undertook to burn out an aching tooth pulp by inserting the head of a match in the cavity and igniting it with a heated wire. As a consequence he was frightfully burned.—*Digest.*

Glycerine Solution of Formalin.—One to 4 per-cent solutions of formalin in glycerine are valuable disinfectant applications in aphthous and parasitic stomatitis, a single application being sufficient for a cure. It is also valuable in parasitic skin troubles. The glycerine counteracts the irritating action of the formalin.—*Ohio Journal.*

The Peridental Membrane and Crowns.—Dr. Black says that more care should be taken in setting crowns, not to injure the peridental membrane. Many a tooth is seriously injured by unwarranted injury to the gum margin and peridental membrane. The tooth is made lame, or worse, and the patient looses the necessary free use of all the teeth in its immediate vicinity.—April *Cosmos.*

Removal of Devitalized Pulp.—If there is reason to believe that minute fragments of pulp tissue remain near the apex, dry the canal and fill with a 25 percent solution of hydronaphthol in alcohol, and with unvulcanized rubber exert enough pressure to saturate the remaining tissue. Then dry the canal, moisten with eucalyptus, and fill with gutta-percha points. — Harold Clark, *Dominion Dental Journal*.

Adapting Metal Backing to Porcelain Facings.— A semi-hard rubber block with holes for the admission of the tooth pins is provided. The backing is applied to the facing approximately, and then placed on the rubber block with the pins inserted in the holes in the rubber. Another piece of semi-hard rubber is then placed on the porcelain and struck with a mallet until the desired adaptation of the backing is obtained.—A. E. Peck, in *Dental Review*.

A New Anesthetic Mixture.—Dr. Wohlgemuth, of Berlin, uses a mixture of oxygen and chloroform, for producing safe and quiet anesthesia. Oxygen is taken from the liquid in the cylinder and as it vaporizes and is being carried through a "face-piece," chloroform is mixed with it by dropping it into the vapor. The narcosis is quiet, the pulse slow and moderate, and recovery rapid. No detrimental results have been noted so far.—*Zahnærztliche Rundschau*.

Four Years' Course in Dentistry in Ontario.— At the regular annual meeting of the Board of Directors of the Royal College of Dental Surgeons of Ontario, April, 1901, the following very important resolution was passed:

*Resolved*, That from and after the first day of May, A.D. 1902, the course in dentistry, in the Province of Ontario, shall be four years' continuous pupilage under indentures, subsequent to matriculation, and, during that period, attendance at four sessions of the School of Dentistry of the Royal College of Dental Surgeons of Ontario.

TO REPAIR BROKEN MODELS.—Use Diamond Magic Invisible Cement, put up in small vials by the Diamond Ink Manufacturing Company, Milwaukee, Wis. It is a clear liquid, and models may be repaired so that the break will hardly be visible. Broken impressions may also be put together in the same way. The cement thickens with age and exposure, and I know of no way to thin it, but it costs only ten cents a bottle, enough to repair two or three hundred models, so that is a small item. Models repaired in this way will stand the pressure of closing the flask and vulcanizing very nicely.—F. H. Roberts, in *Dental Brief*.

THE YOUNG MAN IN DENTISTRY.—He gets old too fast, before middle life he looses enthusiasm and drops out of the race; I should like to hear some one discuss the old and enthusiastic men in dentistry. —*Wright*. Young men should not be less practical, but more studious and scientific.—*Heise*. The dental student gets his education too easily and much of it is technical, and it is therefore not strange that he is not a scientist or a writer.—*Rauh*. The leading workers in the dental profession today are not young men, but old and busy men, too, and they are not necessarily men of great advantages in literary training in early life.—*Smith*.

